# Changes in influenza-associated excess mortality in China between 2012–2019 and 2020–2021: a population-based statistical modelling study

**DOI:** 10.1186/s40249-025-01323-7

**Published:** 2025-06-20

**Authors:** Xiaowei Deng, Jiangmei Liu, Minghan Wang, Nana Chen, Feiran Hao, Juan Yang, Maigeng Zhou, Hongjie Yu

**Affiliations:** 1 Shanghai Institute of Infectious Disease and Biosecurity, School of Public Health, Fudan University, Shanghai, China; 2 Department of Epidemiology, National Vaccine Innovation Platform, School of Public Health, Nanjing Medical University, Nanjing, China; 3 National Center for Chronic and Noncommunicable Disease Control and Prevention, Chinese Center for Disease Control and Prevention, Beijing, China; 4 Key Laboratory of Public Health Safety, Ministry of Education, Fudan University, Shanghai, China

**Keywords:** Influenza-associated excess mortality, Generalized additive model, Excess respiratory mortality, All cause death, Pneumonia and influenza, China

## Abstract

**Background:**

The seasonal cycle of the influenza virus causes substantial morbidity and mortality globally. The impact of severe acute respiratory syndrome coronavirus 2 (SARS-CoV-2) on the circulation of influenza viruses can influence influenza-associated excess mortality. Given the few studies that have explored this topic, the objective of this study was to evaluate influenza-associated excess mortality in the Chinese mainland from 2012 to 2021 and quantify the changes from 2020 to 2021 compared with 2012–2019.

**Methods:**

Using data from national influenza surveillance report and disease surveillance points, we fitted a generalized additive model on all-cause (AC), pneumonia & influenza (P&I), and respiratory (R) mortality rates. In this model, we included data of influenza activity (A/H1N1, A/H3N2 and B), temperature, absolute humidity, the COVID-19 pandemic, and time trends. The excess mortality was estimated by subtracting the fitted baseline mortality from the predicted mortality, which set influenza activity to zero.

**Results:**

The respiratory mortality model explained more than 90% of the variance, indicating the good performance. We found that the influenza-associated mortality was generally decreasing from 2020 to 2021, for instance, influenza A/H1N1-associated excess respiratory mortality (ERM) decreased from 2.62 per 100,000 persons (95% confidence interval: 0.16–5.21) to 0.31 (0.02–0.60) in the northern region and from 3.79 (0.09–7.05) to 0.24 (0.02–0.46) in the southern region between 2012–2019 and 2020–2021. A similar pattern was observed for A/H3N2-associated ERM. While the influenza B remained similar scale, for instance, the ERM was 2.90 (0.72–4.3) and 2.26 (1.76–2.76) in the southern region between 2012–2019 and 2020–2021, respectively. Distinct pattern was observed for the AC and P&I outcomes.

**Conclusions:**

The COVID-19 pandemic has reduced influenza-associated excess mortality, which may be a result of the reduced activity of the influenza virus caused by nonpharmaceutical interventions. Different patterns of regional differences differed for influenza-associated AC, P&I and R mortality. It should be noticed that the contribution of influenza B was generally similar when comparing 2012–2019 and 2020–2021, which highlighted the attention on the influenza B activity. Additional studies are needed to explore the changes in influenza-associated excess mortality afterwards.

## Background

Influenza is an acute respiratory infectious disease caused by influenza viruses, which causes seasonal epidemics and results in substantial morbidity and mortality [1]. Iuliano et al. estimated that annual seasonal epidemics of influenza cause 290,000–650,000 respiratory fatalities worldwide [2]. Deaths caused by influenza infections are significantly underestimated because of the lack of laboratory confirmation [3]. Consequently, influenza-associated (excess) mortality is frequently categorized as a proxy for influenza severity, given the probable contribution during the flu season [4]. Statistical models are commonly used to estimate the excess mortality related to influenza, which enhances our understanding of the full impact of the mortality of influenza [5].

Our previous study evaluated influenza-associated excess respiratory mortality (ERM) in the Chinese mainland from 2010 to 2015 revealing substantial excess respiratory mortality associated with influenza [3]. However, the global COVID-19 pandemic strongly influenced the activity of influenza, which could reshape its burden. The COVID-19 pandemic has caused historically excess death [6, 7]. However, only one study conducted in Denmark revealed a 3.2 (95% confidence interval: 1.1–5.4) per 100,000 person-year excess mortality attributable to influenza during the 2019/20 influenza season [8] Additionally, limited researches in China have been conducted to estimate the excess mortality associated with influenza after 2020 [9].

To fill this gap, this study employed a generalized additive model to quantify excess all-cause, pneumonia & influenza (P&I), and respiratory mortality related to influenza, accounting for the COVID-19 pandemic, meteorological factors, and the inherent time trend from 2012 to 2021 in China. This study systemically analyzed influenza-associated excess mortality in the northern and southern regions of China from 2012 to 2019 and during the COVID-19 pandemic (2020–2021) to provide information on the changing excess mortality contributed by influenza from 2012 to 2021.

## Methods

### Sources of data

Daily mortality and population data from 2012 to 2021 were obtained from the Disease Surveillance Points (DSPs) system [7]. The system consists of 161 DSPs across 31 provincial-level administrative divisions (PLADs) in China, covering 77 million people (6% of the whole population in China). A cleaning procedure was conducted for all-cause mortality to check the reporting status, long-trend stability of mortality, and short-trend stability of each DSP. In addition to all-cause mortality, deaths from pneumonia and influenza (J10–J18) as well as respiratory disease (J00–J99) were recorded according to the International Classification of Diseases, 10th Revision (ICD-10) [10]. All the cleaned data were then aggregated by ISO week and region (the northern and southern) which was consistent to influenza data according to the influenza surveillance protocol [11].

To better understand the representativeness of DSPs system, DSPs data were compared to the census data and nationwide county survey data [12], and it was showed that the DSPs system had good representativeness.

Data of influenza activity were collected via influenza surveillance from the Chinese Weekly Influenza Surveillance Report (CWISR) published by the Chinese National Influenza Center (CNIC). The influenza-like illness (ILI) percentage and influenza-positive rate by type/subtype were checked, and the missing data of the 8th week of 2020 was linearly imputed by data in nearby weeks. Two proxies for influenza activity were used: the influenza-positive rate (number of positive samples/number of samples tested) and the ILI percentage (number of ILIs/number of total outpatients) multiply the influenza-positive rate. We compared both proxies using linear correlation analysis and found that the influenza-positive rate was the better proxy to be included in the analysis. Moreover, we checked the stability of the surveillance, and the number specimen collected and tested was comparable between 2012–2019 and 2020–2021.

As temperature and humidity are important drivers of the seasonality of mortality [13, 14], we include them as covariates to remove the impact on mortality. The daily average ambient temperature and dew temperature by station were collected from the U.S. National Centers for Environmental Information. To calculate the relative humidity, a formula consisting of temperature and dew temperature was employed [15, 16]. The daily temperature and relative humidity were finally aggregated into weeks and regions.

### Statistical analysis

Before the excess mortality was estimated, the linear correlations between mortality and metrics, including influenza activity, temperature, absolute humidity, and relative humidity with a maximum 10-week lag, were explored. To estimate cause-specific excess mortality associated with influenza from 2012 to 2021, a generalized additive model (GAM) with proxies for influenza activity, ambient temperature, and absolute humidity, both before and during the COVID-19 pandemic, as well as time, was employed. The GAM model was separately fitted for the norther and the southern region. The following model formula was applied:

$$\text{E}\left({M}_{t}\right)=\alpha +{\beta }_{1}{Flu}_{h1, t-lag}+{\beta }_{2}{Flu}_{h3,t-lag}+{\beta }_{3}{Flu}_{b,t-lag}+{\beta }_{4}COVID+ns\left({Temp}_{t-lag},df\right)+ns\left({AH}_{t-lag},df\right)+ns\left(t,df\right)$$

In this formula, $\text{E}\left({M}_{t}\right)$ denotes the expected mortality rate at week $t$, which includes three causes of specific mortality, namely, all-cause, P&I, and respiratory. ${Flu}_{h1}$, ${Flu}_{h3}$, and ${and Flu}_{b}$ denote the influenza-positive rates for influenza A/H1N1, A/H3N2, and B, respectively. $COVID$ represents a dummy variable for indicating the time before or during the COVID-19 pandemic (before or after 31 December 2019). $ns(Temp)$, $ns(AH)$ and $ns(t)$ represent the natural cubic splines used to account for the nonlinear relationships between calendar time, temperature, absolute humidity, and mortality rates. The degrees of freedom were set to 3 multiplied by the number of influenza seasons. To account for the lag effects of influenza activity, temperature, and relative humidity, the combination of each variable with a maximum lag of 4 weeks was explored, and the model with the lowest Akaike information criterion (AIC) was selected as the best-fit model, which was recognized as the main result.

Using the model, the predicted mortality rate was estimated and subtracted from the predicted value without the circulation of influenza by setting the influenza activity to zero. For example, if we denoted the mortality rate as Y and the influenza activity as x, E(Y|x = observation)–E(Y|x = 0) was used to calculate the excess mortality. To obtain the confidence interval, a residual bootstrap with 1000 replications was implemented.

All the statistical analyses were performed using R software (version 4.2.2; R Foundation for Statistical Computing, Vienna, Austria; https://www.r-project.org/), and results were reported as the average and 95% *CI*.

## Results

**Fig. 1:**
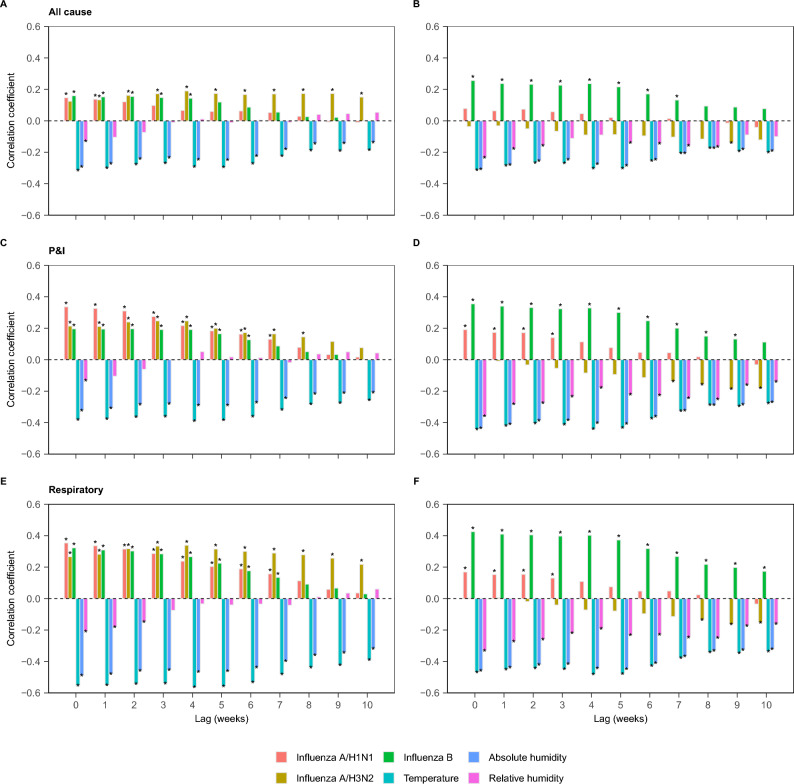
Lag correlation with mortality for different lags. The left column refers to the northern region (**A**, **C**, **E**), and the right column refers to the southern region (**B**, **D**, **F**). *P&I* pneumonia and influenza

The lagged correlation analysis revealed that the intensity of influenza virus activity, temperature, absolute humidity, and relative humidity, including all-cause, P&I, and respiratory factors (except for influenza A/H3N2 in the southern region; Fig. 1), were significantly correlated with mortality. A stronger correlation was detected for respiratory death in both regions. For the northern region, influenza A/H3N2 showed a significant lagged correlation (i.e., the coefficient increased from 0.27 to 0.33 for respiratory in the northern region, with lag increased from 0 to 3, and the *P*-values were all < 0.001). The coefficients were generally similar among different lags, whereas they were greater for deaths related to influenza A/H1N1 and P&I. For the southern region, influenza B showed a greater positive correlation (i.e., the correlation is over 0.4 for respiratory with lag 0 in the southern, while the correlation is 0.32), whereas influenza A/H3N2 was negative but non-significant (i.e., for P&I with lag 0, the coefficient is -0.15 in the southern region and the *P*-value is 0.94, while in the northern region, the coefficient is 0.21 and the *P*-value is < 0.001). All the meteorological factors were negatively correlated with death, except for absolute humidity, which showed a relatively high correlation.

We selected the best-fit model with the minimum AIC for the northern and southern regions (Table 1). For the three types of cause-specific mortality, the variation (*R*^2^) ranged from 76 to 93%. The model for the southern region showed an 85% variation, whereas the model for the northern region showed an 87% variation in terms of P&I, slightly better than that for the southern region. Moreover, the lags achieved great variation among the three outcomes and regions. For all-cause mortality in the northern region, the lags of influenza A/H1N1, influenza A/H3N2, influenza B, temperature, and absolute humidity were 4, 4, 0, 1, and 1 weeks, respectively. For all-cause mortality in the southern region, the lags were 4, 1, 3, 1, and 0 weeks, respectively. In the northern region, influenza B for all-cause and respiratory mortality and influenza A/H3N2 for P&I mortality varied, whereas in the southern region, the lag variation was greater.

**Table 1:** The best fit model information by region and mortality

Region	Outcome	Lag (week)					AIC	R-squared
		Influenza A/H1N1	Influenza A/H3N2	Influenza B	Temperature	Absolute humidity		
North	All-cause	4	4	0	1	1	1390.69	0.80
North	P&I	2	1	4	3	1	2625.74	0.87
North	Respiratory	3	4	1	3	1	1577.44	0.93
South	All-cause	4	1	3	1	0	1397.26	0.76
South	P&I	3	2	4	1	0	2805.97	0.85
South	Respiratory	2	2	4	1	0	1942.67	0.91

*AIC* Akaike information criterion, *P&I* pneumonia and influenza

**Fig. 2:**
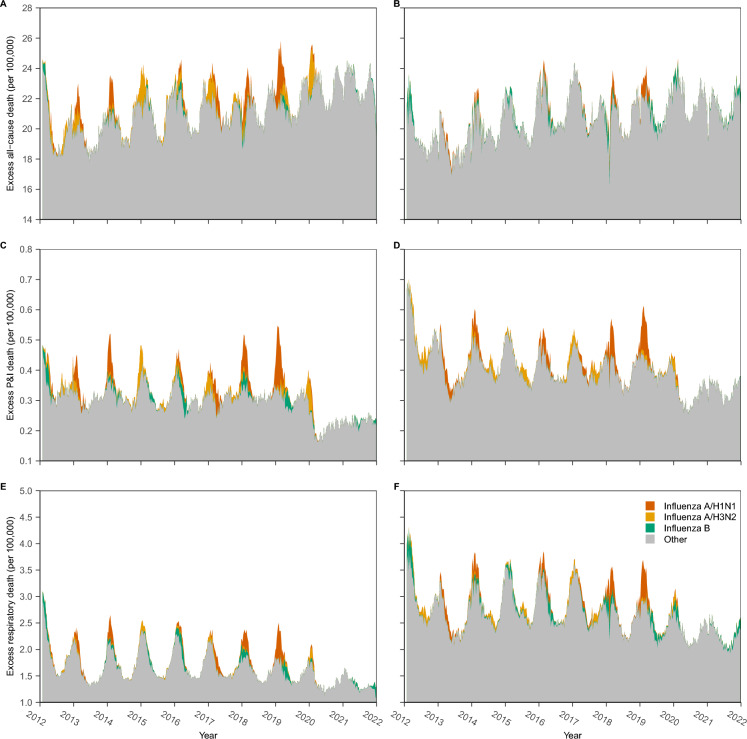
Contribution of mortality from 2012 to 2021. The left column refers to the northern region (**A**, **C**, **E**), and the right column refers to the southern region (**B**, **D**, **F**). *P&I* pneumonia and influenza

On average, all-cause mortality was higher in the northern region than in the southern region, whereas P&I and respiratory mortality were higher in the southern region than in the northern region (Fig. 2). In the northern region, influenza activity significantly contributed to mortality during the winter, whereas almost no contribution was observed during the summer (i.e., the excess respiratory mortality in the northern region is 37.1 per 100,000 persons on average during 125 months, while it is 3.1 per 100,000 persons on average during 611 moths). By contrast, in the southern region, especially for respiratory mortality, a significant contribution was observed in the summer (i.e., the excess respiratory mortality in the southern region is 49.1 per 100,000 persons on average during 125 months, while it is 16.9 per 100,000 persons on average during 611 moths). A significant reduction in P&I and respiratory mortality was observed in both regions when comparing 20,122,019 and 20,202,021; however, the reduction was lower in the southern region than that in the northern region.

The influenza-associated excess all-cause and P&I mortalities in the northern region were higher than those in the southern region (i.e., 25.29 per 100,000 persons vs 4.61 per 100,000 persons for all-cause, and 0.81 per 100,000 persons vs 0.69 per 100,000 persons for P&I in the northern and southern regions, respectively, in 2015; Table 2), whereas the influenza-associated ERM was higher in the southern region than in the northern region (i.e., 2.69 per 100,000 persons vs. 5.34 per 100,000 persons for respiratory in the northern and southern regions, respectively, in 2015). Moreover, the all-cause excess mortality related to influenza varies highly from that related to P&I and respiratory mortality among seasons. The years 2014, 2017–2019 had higher influenza-associated excess mortalities, which were 31.47, 40.69, 30.38, and 49.23 per 100,000 persons for all causes; 1.89, 1.71, 1.88, and 2.86 per 100,000 persons for P&I; and 5.02, 5.00, 5.95, and 8.68 per 100,000 persons for respiratory disease, respectively.

**Table 2:** Influenza-associated mortality in different seasons

Season^a^	Influenza associated excess all-cause mortality (per 100,000 persons)		Influenza associated excess P&I mortality (per 100,000 persons)		Influenza associated excess respiratory mortality (per 100,000 persons)	
	North	South	North	South	North	South
2012	25.07	7.82	1.53	0.97	4.28	7.91
2013	24.8	7.19	1.11	1.21	3.61	6.08
2014	31.47	10.82	1.89	1.42	5.02	8.15
2015	25.29	4.61	0.81	0.69	2.69	5.34
2016	24.71	17.24	1.51	1.28	4.8	8.51
2017	40.69	6.92	1.71	1.23	5	6.73
2018	30.38	22.52	1.88	1.52	5.95	10.29
2019	49.23	20.55	2.86	1.99	8.68	12.11
2020	22.28	3.48	0.65	0.33	2.14	2.99
2021^b^	4.78	9.19	0.24	0.03	1.68	2.79

*P&I* pneumonia and influenza

^a^The season runs from the first week of October of the year to the last week of September of the next year

^b^The data for the 2021 season were incomplete, and the data were limited to December 2021

**Fig. 3:**
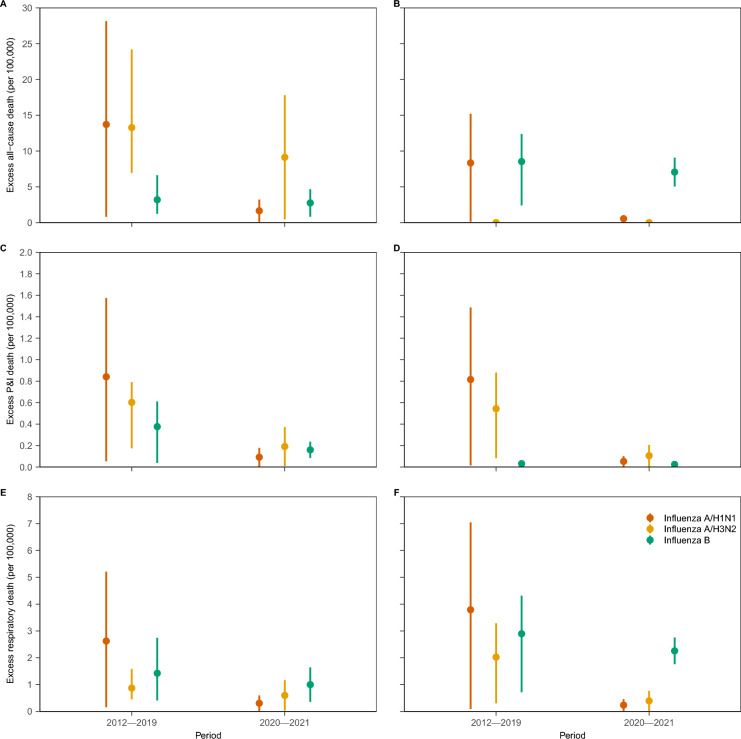
Influenza-associated mortality during 2012–2019 and 2020–2021 by influenza subtype. The left column refers to the northern region (**A**, **C**, **E**), and the right column refers to the southern region (**B**, **D**, **F**). *P&I* pneumonia and influenza

For both regions, influenza A/H1N1 was the most important contributor of excess mortality from 2012 to 2019, accounting for 13.72 (95% *CI*: 0.81–28.15), 0.84 (95% *CI*: 0.05–1.57), and 2.62 (95% *CI*: 0.16–5.21) per 100,000 persons for all-cause, P&I, and respiratory diseases, respectively, in the northern region and 8.34 (95% *CI*: 0.13–15.21), 0.82 (95% *CI*: 0.02–1.49), and 3.89 (95% *CI*: 0.09–7.05) per 100,000 persons for all-cause, P&I, and respiratory diseases, respectively, in the southern region (Fig. 3). Influenza B caused a generally greater proportion of excess all-cause and respiratory mortality in the southern region than in the northern region (i.e., 3.20 vs 8.53 for all-cause mortality in the northern and southern regions and 1.42 vs. 2.90 for respiratory mortality in the northern and southern regions, respectively).

A significant decrease in excess mortality due to influenza A can be observed from 2020 to 2021 compared with the average level from 2012 to 2019 (Fig. 3). For example, the influenza A/H1N1-related excess all-cause mortality decreased from 2.62 (95% *CI*: 0.16–5.21) to 0.31 (95% *CI*: 0.02–0.60) per 100,000 persons in the northern region and from 3.79 (95% *CI*: 0.09–7.05) to 0.24 (95% *CI*: 0.02–0.46) per 100,000 persons in the southern region. However, for influenza B, the related excess mortality rate remained relatively stable. The ERM contributed by influenza B were 1.42 (95% *CI*: 0.41–2.74) and 1.00 (95% *CI*: 0.36–1.64) per 100,000 persons in the northern region and 2.90 (95% *CI*: 0.72–4.31) and 2.26 (95% *CI*: 1.76–2.76) per 100,000 persons in the southern region.

## Discussion

Influenza activity and meteorological factors, including temperature and humidity, often influence mortality from exposure until a certain lag period. The correlation analysis revealed that the regional pattern differed for each factor, especially for influenza activity. The variation in the lagged correlation was observed among influenza A/H1N1, A/H3N2 and B, with a greater correlation for influenza B in the southern region. The significance of influenza A/H1N1 for P&I and respiratory mortality in the southern region only appeared with a maximum lag of 4 weeks (approximately one month). For meteorological factors, a strong negative correlation between temperature/humidity and mortality was detected and was generally stable within a 4–5-week lag. This observation is similar to the findings of a study conducted by Chen et al. [13]. In this study, mortality was asymmetrically associated with temperature, which indicated a greater correlation with cold weather, whereas extremely hot weather was also related to a greater risk of mortality. Extreme cold weather can influence mortality after more than 2 weeks. The effect of temperature on mortality can last for one month. This information indicates the rationale for the selection of a maximum of 4 week lag combinations when the model selection procedure is conducted, [17] which is different from our previous work [3].

The best-fit model for estimating excess all-cause, P&I, and respiratory mortality had distinct lag combinations and varied based on region. A relatively longer lag effect was chosen for influenza B than for influenza A. This finding is consistent with the study by Li et al. [18] In terms of trends, a longer lag effect was found for influenza B/Victory, which was 5–13 days. The inconsistency of the scale for the lag effect for influenza virus may be caused by the resolution of time. Our study was conducted based on weekly aggregated data rather than daily data using spline methods. Even if the detailed temporal resolution could have provided more information and knowledge, the convention beyond the surveillance rationale could be problematic. Moreover, Lytras et al. [17] also reported that the lag effect varies across different age groups.

The estimation of average annual influenza-associated excess all-cause mortality from 2012 to 2019 was similar to a previous report conducted in Guangzhou from 2015 to 2018 (25.06 per 100,000 persons, 95% *CI*: 19.85–30.16) [18] and Shanghai from 2010 to 2015 (27.66 per 100,000 persons, 95% *CI*: 22.3–33.88) [19]. Liu et al. estimated that 9.9 per 100,000 persons (95% *CI*: 6.28–13.52) excess all-cause mortality in Hefei from 2010 to 2015 [20], which is relatively lower than what we estimated in the southern region. This reveals inner-regional differences, which is well illustrated in our previous work [3]. Moreover, Feng et al. estimated that the average annual influenza-associated excess all-cause mortality ranged from 3.4 to 32.7 in three northern cities and 3.5–17.4 in five southern cities from 2002 to 2008 [10]. A similar pattern for the northern and southern regions was detected in our study after the 2009 H1N1 pandemic, and the results varied for each influenza season. The higher burden in the southern region may be related to the different circulation pattern of influenza. As our previous study, the influenza virus tends to show a twin-peak pattern per year in the southern region, and even a whole year circulation can be observed in some most southern provinces.

For influenza-associated excess P&I and respiratory mortalities, we estimated that P&I should be lower than respiratory mortality and that respiratory mortality should be lower than all-cause mortality. This phenomenon is intrinsic and has also been reported in several prior studies. For instance, Liu et al. modeled the excess mortality in Hefei from 2010 to 2015 and reported that the excess respiratory mortality related to influenza was 2.74 per 100,000 persons (95% *CI*: 1.87–3.5), whereas the ERM related to influenza was 0.28 for all causes, and the P&I mortality was 0.55 per 100,000 persons (95% *CI*: 0.19–0.92), which was 0.2 for respiratory mortality [20]. Our estimation of ERM was greater than that in Hefei, ranging between 2.69 and 8.68 in the northern region and between 5.34 and 12.11 in the southern region from 2012 to 2019. This finding confirms our previous work, which indicated that ERM ranged from 1.7 to 13 per 100,000 persons across provinces in China [3]. Unlike all-cause mortality, the ERM is greater in the southern region than in the northern region. This finding is consistent with our previous estimates, where the average excess respiratory rate was 8.1 in three southern provinces (Fujian, Guangxi, and Guangdong) and 3.6 in three northeastern provinces (Heilongjiang, Liaoning, Jilin) [3]. Our estimated excess P&I mortality ranged from 0.69 to 2.86 from 2012 to 2019, which was higher than that estimated in Shanghai (0.34 per 100,000 persons, 95% *CI*: 0.16–0.58) [19] but lower than that reported in Guangzhou (3.88 per 100,000 persons, 95% *CI*: 2.8–4.84) [18].

Since the COVID-19 pandemic, an increasing number of studies have indicated a surge in COVID-19 as well as control measures (nonpharmaceutical interventions [NPIs]) implemented globally, which significantly influence influenza activity [8, 21–23]. Our results indicated that the low intensity of circulation of the influenza virus may be caused by NPIs, subsequently causing low excess mortality related to influenza. The all-cause mortality decreased from an average of 21.83 to 9.93, the P&I decreased from an average of 1.48 to 0.31, and the respiratory mortality decreased from 6.57 to 2.4. Nielsen et al. reported that the excess all-cause mortality attributed to influenza during the 2019–2020 influenza season was mild (3.2 per 100,000 persons, 95% *CI*: 1.1–5.4) [8]. These findings indicated that the excess mortality burden was lower than that in the previous season. This downward trend is similar to that in our study, but the scale is lower.

Moreover, we found a distinct decreasing pattern of excess mortality for influenza types/subtypes. A significant reduction in excess all-cause, P&I and respiratory mortality was observed for influenza A/H1N1 and A/H3N2 (i.e., for ERM in the northern region, influenza A/H3N2 caused 2.02 vs 0.39 per 100,000 persons on average from 2012 to 2019 and 2020–2021, respectively), whereas influenza B remained at a similar scale (i.e., for excess respiratory mortality in the southern region, influenza B contributed to 2.9 vs. 2.26 per 100,000 persons from 2012 to 2019 and 2020–2021, respectively). Our study revealed the changing pattern of influenza-related excess mortality after the COVID-19 pandemic. Notably, the influenza B/Victoria lineage was mostly detected in the African Region and Western Pacific Region from 2020 to 2021, and influenza B/Yamagata surprisingly disappeared during the COVID-19 pandemic [24]. The relatively high activity of influenza B/Victoria could possibly lead to the high excess mortality burden.

The study also revealed that the influenza-associated excess mortality burden was greater in Chinese mainland than in Republic of Korea and the U.S. Park et al. estimated that the excess all-cause mortality was 5.97 per 100,000 persons (95% *CI*: 4.89–7.19) in Republic of Korea from 2003 to 2013 [25]. In the U.S., Hansen et al. estimated that the excess all-cause mortality associated with influenza was 9 per 100,000 persons (95% *CI*: 8.3–9.7) and that its association with respiratory disease was 3.4 per 100,000 persons (95% *CI*: 3.2–3.5) from 1999 to 2018 [26]. This could have been caused by increased influenza vaccine coverage. Seo et al. reported that total influenza vaccination coverage ranged from 38 to 44.1% from 2005 to 2014 in Republic of Korea [27] while in the U.S., influenza vaccination coverage among adults increased from 38.3% in the 2010–2011 season to 43.4% in the 2015–2016 season [28], with influenza vaccination during pregnancy increasing from 8.8% to 50.9% in 2002–2012 [29]. Moreover, a meta-analysis concluded that vaccination coverage among the general population was 9.4% in China [30], which was a quarter to one-third of that in Republic of Korea, confirming that influenza vaccine coverage in China was low with very little awareness among the population.

For influenza-associated excess mortality from 2020–2021, only Nielsen et al.[8] reported 3.2 per 100,000 person-years (95% *CI*: 1.1–5.4) in the 2019/20 season in Denmark, and the mortality attributable to COVID-19 from the 2020 week of 9 to 2020 week of 20 was estimated to be 16.2 per 100,000 person-years (95% *CI*: 12.0–20.4). Our estimation is marginally greater, which may be caused by the different situations of COVID-19 as well as the various measures taken. And one study utilizing the negative binomial regression model without including meteorological factors to estimate multi-cause mortality linked to influenza virus infection 2012–2021 [9]. The study estimated the average of annual excess all-cause mortality was 14.53 per 100,000 persons (95% *CI*: 9.12–17.59) and the respiratory mortality was 3.73 per 100,000 persons (95% *CI*: 2.33–4.43), while not reported the estimates from 2020–2021.

This study has several limitations. First, the mortality data was not available for influenza-season after 2021; hence, the changing pattern could not be explored after adjustment for COVID-19 control measures in China since late 2022. The influenza-associated mortality after 2022 merits further study. Second, the analysis was based on region rather than provincial administration. The detailed geographical resolution could be more informative, whereas aggregated regional analysis may decrease the influence of the surveillance of influenza activity as well as death by sporadic disease caused by COVID-19 from 2020 to 2021. Third, the age-specific excess mortality was not estimated because we had no age information on influenza activity. Fourth, the study did not include the activity of other respiratory pathogens (i.e., respiratory syncytial virus), which may contribute to excess mortality and cocirculate with influenza. The study included time trends as well as meteorological factors, which can partially reflect these influences in the absence of real surveillance. We strongly recommend systemic pathogenic monitoring through symptom-based surveillance and making it publicly available.

## Conclusions

Our study revealed a substantial mortality burden associated with influenza in China. Compared with the 2012–2019 period, the COVID-19 pandemic strongly reduced the excess mortality related to influenza, while the observed ongoing surge in influenza virus activity indicated a potentially greater impact and burden of influenza since that time period. And it should be noticed that the contribution of influenza B was generally similar when comparing 2012–2019 and 2020–2021, which highlighted the attention on the influenza B activity. Moreover, the relatively high excess mortality associated with influenza may be due to low vaccination coverage in China. Thus, it is important to promote awareness and address current related policies on influenza vaccination.

## Data Availability

All influenza data is publicly available through querying the official website of National Influenza Center (China), and all DSPs data should contact our corresponding authors.

## Abbreviations

AC: All-cause
AIC: Akaike information criterion
COVID-19: Coronavirus disease 2019
DSP: Disease surveillance point
ERM: Excess respiratory mortality
GAM: Generalized additive model
ICD-10: International classification of diseases, 10th revision
ILI: Influenza-like illness
NPI: Nonpharmaceutical intervention
PI: Pneumonia and influenza
R: Respiratory
SARS-CoV-2: Severe acute respiratory syndrome coronavirus 2
